# Evaluation of Radicular Dentin Thickness in Maxillary and Mandibular Central Incisor, Canine, and Premolar Teeth

**DOI:** 10.7759/cureus.56974

**Published:** 2024-03-26

**Authors:** Esra Arılı-Öztürk, Mediha Büyükgöze-Dindar

**Affiliations:** 1 Endodontics, Trakya University, Edirne, TUR; 2 Health and Science Vocational School, Trakya University, Edirne, TUR

**Keywords:** tooth root, tooth, endodontics, dentin, dental pulp cavity

## Abstract

Background

This study aimed to compare the radicular dentin thickness in single-rooted maxillary and mandibular anterior and premolar teeth by measuring on four different surfaces (buccal, lingual, mesial, and distal) at three different levels (apical, middle, and coronal).

Methods

A total of 150 single-rooted human anterior and premolar teeth were included in the present study. The teeth were sectioned at the cemento-enamel junction (CEJ; coronal), 4 mm away from the apex (apical), and the midpoint between these two distances (middle). First, the teeth were divided buccolingually into two parts, and the dentin thickness in the mesial/distal region was measured. Subsequently, both parts were divided again to measure the dentin thickness in the buccal/lingual region. All measurements were recorded, and statistical analysis was performed.

Results

Apical radicular dentin thickness was significantly less than CEJ (p < 0.001). The maxillary central incisor, maxillary, and mandibular canine exhibited the maximum radicular dentin thickness, whereas the mandibular premolars showed the minimum. Radicular dentin thicknesses on the lingual and buccal surfaces were significantly higher than those on other surfaces (p < 0.001).

Conclusion

The lowest radicular dentin thickness values were observed in the premolars, especially on mesial and distal surfaces. Considering these areas as danger zones and paying attention during dental procedures are essential to avoid possible complications.

## Introduction

Root and canal morphology vary according to the type and structure of the teeth. Therefore, understanding radicular anatomy is crucial for successful root canal treatment or post-space preparation. Insufficient information about radicular dentin thickness can lead to perforations or vertical root fractures during canal instrumentation or post-replacement procedures. In particular, endodontic treatment becomes challenging in teeth with difficult-to-detect extra canals and complex root and canal morphology [1]. Therefore, excessive dentin tissue loss may occur during the preparation of the access cavity and detection of the root canal orifices, or excessive enlargement of the root canals during instrumentation may reduce the fracture resistance of the tooth [2]. In addition, the amount of residual radicular dentin has a vital role in placing posts to restore the endodontically treated teeth with excessive tissue loss [3]. Accordingly, performing endodontic treatment with knowledge about the radicular dentin thickness in teeth with different structures is essential.

Danger zones are defined as areas of the root canal walls where the dentin is consistently thin (especially where grooves are present or between two fused roots), predisposing the teeth to mechanical perforation during endodontic treatment [4,5]. In these regions, which already have thin dentin walls, excessive loss of dentin structure caused by over-instrumentation during canal shaping or post-space preparation may lead to perforations or root fractures due to exposure to functional loads [6]. It is known that the fracture resistance of endodontically treated teeth decreases as the amount of residual dentin decreases. Furthermore, the internal tension increases as the diameter of the post expands. For this reason, the diameter of the applied post should not exceed one-third of the total root width and should be surrounded by a dentin wall of at least 1 mm [7]. Therefore, a comprehensive understanding of these danger zones is necessary to avoid situations that could compromise the clinical fate of teeth during canal shaping or post-space preparation procedures [8].

There are very few studies in the literature about the radicular dentin thickness of various teeth [7-9]. In these studies, researchers either investigated the morphology of the root canal system in mandibular incisors according to the number of root canals or analyzed the characteristics of the dangerous areas and the resulting clinical outcomes in mandibular and maxillary molars. This study aims to determine the danger zones by measuring the dentin and cementum thickness of all surfaces around the pulp chamber of single-rooted anterior and premolar teeth at the apical, middle, and coronal levels.

## Materials and methods

This study was carried out after obtaining ethical permissions from the Trakya University Non-interventional Scientific Research Ethics Committee (TÜTF-GOBAEK 2023/378), and informed consent forms were obtained from all patients before extraction of the teeth to be used. A total of 150 human teeth extracted for orthodontic and periodontal reasons were included in the study. After removing the calculus with an ultrasonic scaler, the teeth were stored in a buffered formalin or 2% sodium hypochlorite solution to remove remnants of the periodontal ligament. Single-rooted maxillary and mandibular central, canine, and premolar teeth without any caries, cracks, or fractures were included in the study (n:25). Conversely, teeth with unformed apices, root resorption, and those treated with root canal filling, posts, or crown restoration were excluded from the study. Furthermore, teeth with complex root canal morphology and calcifications that could not be identified as single canals after sectioning were not included in the study. Hard and soft tissue residues on the surface of the teeth were cleaned. The teeth were dried, sectioned, and prepared for measurement. In maxillary and mandibular teeth, incisors, canines, and premolars groups were formed to equalize the number of groups. Therefore, no distinction was made between mandibular central or lateral incisors and maxillary and mandibular first or second molars. The premolars with a single root and a single canal were included in the same group. Since no distinction was made between maxillary and mandibular central and lateral incisors, maxillary central incisors were chosen as representatives of maxillary incisors, considering the maxillary lateral incisors' size and distal root inclination which makes it challenging to section their roots.

As in a previous study [9], the teeth were marked at the enamel-cementum junction (CEJ) level, 4 mm from the apical and the midpoint between these two distances, and sectioned perpendicular to the long axis of the root. Thus, apical, middle, and coronal triple sections were obtained.

**Figure 1:**
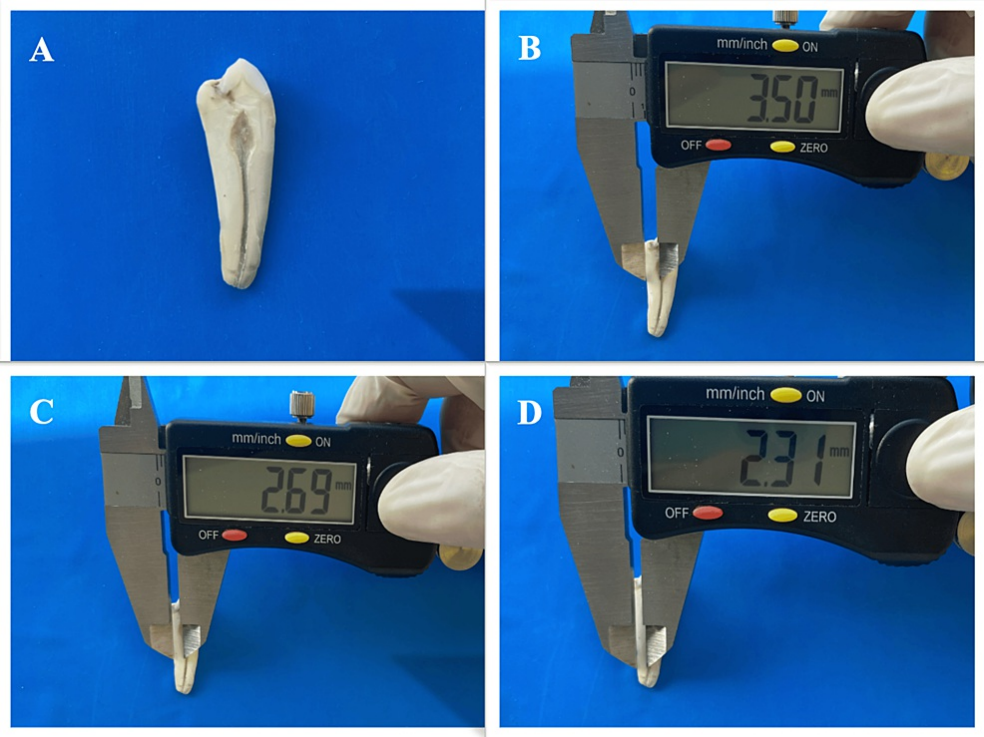
Illustration of buccolingually sectioned premolar teeth (A) and the measurement of lingual side dentin thickness using a precision mechanical caliper at the cemento-enamel junction (CEJ) (B), middle (C), and apical parts (D)

Initially, the teeth were sectioned buccolingually with a 0.2 mm thick ultra-thin silicon carbide separator disk, passing through the middle of the pulp chamber and forming the buccal and lingual portions. Afterward, the thickness of the buccal and lingual sides was measured using a precision mechanical caliper, from both parts of the root canal (Figure 1). Subsequently, both parts were sectioned again to obtain mesial and distal portions with a separator disk passing through the middle of the pulp chamber. After the mesial and distal dentin thickness measurements were made on the obtained sections, all measurements were repeated in apical, middle, and coronal portions for each tooth. All measurements were made by a single operator (MBD) for standardization. The obtained data were recorded, and statistical analysis was performed.

Statistical analysis

With the power analysis conducted before the study (%95 power, α:0.05 probability error, effect size: 0,284) [9], it was determined that 25 samples should be included in each group.

Intraobserver agreement level was defined by reliability analysis. Intraclass correlation coefficients (ICCs) and confidence intervals (CIs) were calculated based on repeated measurements of randomly selected 10% of the samples by the same operator at different times.

The conformity of the data to the normal distribution was checked with skewness, kurtosis, and Shapiro-Wilk tests. Kruskal-Wallis test was used for intergroup comparisons of non-normally distributed data, and Mann-Whitney U tests were used for pairwise comparisons. Friedman and Wilcoxon tests were used for intra-group comparisons. Arithmetic mean and standard deviation were given as descriptive statistics, and the limit of significance was p < 0.05.

## Results

The intraobserver agreement analysis yielded a satisfactory ICC (p < 0.001). The ICC coefficient for intraobserver agreement was 0.973 (95% CI: 0.96 to 0.98; p < 0.001).

The radicular dentin thicknesses obtained from different parts of the teeth according to the tooth subgroups are given in Table 1. According to Kruskal-Wallis analysis of variance, there were significant differences between dentin thicknesses in various regions (in between CEJ/middle/apical sections) of all teeth without any tooth discrimination (p < 0.001). Radicular dentin thickness in the apical region was significantly less than CEJ.

**Table 1:** The radicular dentin thicknesses obtained from different parts of the teeth according to the tooth subgroups Different capital letters (A-E) indicate significant differences between the same columns, and different lowercase letters (a-c) indicate significant differences between the same rows. CEJ: Cemento-enamel junction (p < 0.05).

Region	Teeth group	N	Mesial	Distal	Buccal	Lingual	p-values
			(Mean ± SD)	(Mean ± SD)	(Mean ± SD)	(Mean ± SD)	
CEJ	Maxillary central incisors	25	2.58 ± 0.4^A,a^	2.48 ± 0.4^A,a^	2.75 ± 0.3^A,a^	3.00 ± 0.3^A,b^	0.000
	Mandibular incisors	25	1.50 ± 0.2^C,a^	1.60 ± 0.3^C,a^	2.33 ± 0.2^D,b^	2.40 ± 0.3^B,b^	0.000
	Maxillary canines	25	2.49 ± 0.3^A,a^	2.38 ± 0.4^A,a^	3.12 ± 0.4^B,b^	3.39 ± 0.4^A,b^	0.000
	Mandibular canines	25	2.31 ± 0.3^A,a^	2.33 ± 0.3^A,a^	2.90 ± 0.3^AB,b^	3.17 ± 0.7^A,c^	0.000
	Maxillary premolars	25	1.90 ± 0.3^B,a^	1.94 ± 0.2^B,a^	2.54 ± 0.3^AC,b^	2.58 ± 0.3^B,b^	0.000
	Mandibular premolars	25	2.00 ± 0.3^B,a^	2.03 ± 0.3^B,a^	2.68 ± 0.2^AC,b^	2.55 ± 0.2^B,b^	0.000
P			0.000	0.000	0.000	0.000	
Middle	Maxillary central incisors	25	2.16 ± 0.3^A,a^	2.06 ± 0.3^A,a^	2.45 ± 0.2^A,b^	2.58 ± 0.2^A,b^	0.000
	Mandibular incisors	25	1.20 ± 0.2^D,a^	1.28 ± 0.2^C,a^	2.24 ± 0.3^C,b^	2.31 ± 0.2^C,b^	0.000
	Maxillary canines	25	1.97 ± 0.3^AB,a^	1.86 ± 0.4^A,a^	2.81 ± 0.3^B,b^	3.17 ± 0.5^B,b^	0.000
	Mandibular canines	25	1.89 ± 0.4^B,a^	1.84 ± 0.3^A,a^	2.76 ± 0.3^B,b^	2.97 ± 0.3^B,b^	0.000
	Maxillary premolars	25	1.45 ± 0.3^C,a^	1.40 ± 0.3^BC,a^	2.49 ± 0.3^A,b^	2.51 ± 0.3^A,b^	0.000
	Mandibular premolars	25	1.60 ± 0.3^C,a^	1.56 ± 0.3^B,a^	2.40 ± 0.3^AC,b^	2.63 ± 0.3^A,b^	0.000
P			0.000	0.000	0.000	0.000	
Apex	Maxillary central incisors	25	1.60 ± 0.3^A,ab^	1.55 ± 0.3^A,a^	1.73 ± 0.2^A,b^	1.93 ± 0.3^A,c^	0.000
	Mandibular incisors	25	0.89 ± 0.2^C,a^	0.91 ± 0.2^C,a^	1.75 ± 0.3^A,b^	1.75 ± 0.3^B,b^	0.000
	Maxillary canines	25	1.36 ± 0.3^BD,a^	1.25 ± 0.3^BD,a^	1.83 ± 0.4^A,b^	2.02 ± 0.3^A,b^	0.000
	Mandibular canines	25	1.38 ± 0.3^BD,a^	1.31 ± 0.3^ABD,a^	2.04 ± 0.3^BC,b^	2.11 ± 0.2^A,b^	0.000
	Maxillary premolars	25	1.20 ± 0.2^B,a^	1.04 ± 0.3^BC,a^	1.91 ± 0.3^ABC,b^	1.88 ± 0.3^AB,b^	0.000
	Mandibular premolars	25	1.17 ± 0.3^BE,a^	1.20 ± 0.3^B,a^	1.84 ± 0.2^AB,b^	1.99 ± 0.3^AB,b^	0.000
P			0.000	0.000	0.002	0.000	0.000

When comparing only the tooth subgroups, significant differences were observed in dentin thickness among maxillary and mandibular incisors, canines, and premolars (p < 0.001). The radicular dentin thickness of the maxillary central incisor as well as the maxillary and mandibular canine was higher than the other tooth subgroups. The thinnest radicular dentin walls were detected at the mandibular premolars, followed by the maxillary and mandibular premolars.

When the investigations were made according to both teeth subgroups and regions, it was discovered that the thickest radicular dentin was present in the lingual wall in every section, followed by the buccal wall. Radicular dentin thickness on the lingual and buccal surfaces was significantly higher than that on the mesial and distal surfaces (p < 0.001).

## Discussion

Dentin loss and root perforations that occur during excessive instrumentation are among the causes that may compromise the prognosis of endodontically treated teeth [10]. Root canal instrumentation and post-space preparation result in a reduction in radicular dentin thickness [11]. Depending on the decreased amount of radicular dentin thickness, the risk of vertical root fracture increases, and the location, direction, and probability of the fracture are directly affected by the thickness of the residual dentin tissue [12]. Moreover, the material and type of the post applied are also effective on the possibility of fracture or perforation. Increased risk of perforation or vertical fracture was reported in previous studies, especially during the preparation and placement of parallel-sided active metal posts [10-13]. Attention should also be paid to danger zones to reduce risks during the preparation and placement of such posts. Therefore, the present study aimed to measure the radicular dentin thickness of single-rooted maxillary and mandibular central incisor, canine, and premolar teeth. Thus, potential risks during endodontic treatment can be predicted and reduced, resulting in improved prognosis.

In teeth with multiple roots and canals, radicular dentin thickness varies considerably and becomes difficult to determine due to the presence of isthmus and complex canal anatomy. Therefore, only single-rooted and single-canal teeth were included in this study to provide standardization. Although central incisors were selected for the maxillary incisors, no central and lateral distinction was made for the mandibular incisors. As reported in the literature, no distinction was made between mandibular incisors in the current study since these two teeth are morphologically very similar [14].

Bellucci and Perrini reported in a study conducted on central incisors and premolar teeth that, similar to the present study, the thickest radicular dentin was observed in the lingual wall, and the thinnest was in the proximal [9]. They also stated decreased radicular dentin thickness toward the apex. Similarly, Khedmat et al. examined the two-rooted mandibular incisors and determined that the lowest radicular dentin thickness was in the proximal walls, and they also reported an increased risk of perforation in the relevant areas [15]. Moreover, since they determined that the lingual wall thickness is greater than the buccal, it has been suggested that the use of the lingual canal may be more appropriate for post-placement [15]. Amardeep et al. [16] examined the morphology of the maxillary and mandibular canines in the Indian population and reported that the thinnest radicular dentin wall was on the mesial surfaces. Consistent with this, in this study, the thinnest radicular dentin walls of the maxillary and mandibular canines were detected on the mesial and distal surfaces.

In a study in which mandibular first premolars with C-shaped canal configurations were analyzed with micro-CT, it was stated that the minimal radicular dentin thickness was observed on the mesial wall of the canal in the middle and apical sections [17]. In the current study, the thinnest radicular dentin was detected on the mesial and distal surfaces of the mandibular premolar teeth. Some Studies evaluating the danger zones of mandibular molars reported that the minimal radicular dentin thickness is located 3 mm below the furcation and within the mesial root [4-6]. Furthermore, it is stated that there is no significant difference between the radicular dentin thicknesses of the mesiobuccal and mesiolingual roots [4,18]. When the radicular dentin thickness of the canals located in the mesial root of maxillary molar teeth was evaluated, it was determined that MB2 canals have thinner mesial and distal radicular dentin walls compared to MB1 [19]. In a study conducted by Heyse et al., it was revealed that the dentin thickness in the distal wall of the MB2 canal of maxillary molar teeth (which is the danger zone for maxillary molar) decreased by 32% after root canal instrumentation [20]. This situation increases the risk of perforation or fracture. In a study in which the residual dentin thickness was measured after post-preparation of the palatal root of the maxillary molar teeth, it was shown that the thinnest radicular dentin wall was on both the buccal and palatal surfaces after the post-space was prepared [21].

Numerous methods have been proposed for the assessment of radicular dentin thickness (e.g., radiographs, serial sectioning, microcomputed tomographic [micro-CT] imaging, and cone-beam computed tomographic [CBCT] imaging). However, it has been disclosed that radiography shows the radicular dentin thicker than it is, and therefore, radiographs should not be considered a reliable method for this purpose [3,11]. Using micro-CT, each tooth area can be evaluated, and more precise results can be obtained. However, micro-CT exposes patients to an extra radiation dose, has a limited sample size, and is an expensive technology [3]. Accordingly, sectioning and measuring sections with a digital caliper was the method of choice for the present study.

This study underscores the significance of radicular dentin thickness variations, emphasizing the vulnerability of premolars, particularly on mesial and distal surfaces, as potential danger zones during dental procedures. Knowledge of these variations is crucial to mitigate complications and enhance clinical outcomes. Despite the insightful findings provided by this study regarding radicular dentin thickness in various tooth subgroups, several limitations must be acknowledged. First, the study focused solely on single-rooted teeth, thereby excluding multi-rooted teeth with potentially different radicular anatomy. This exclusion may limit the generalizability of the results to clinical scenarios involving multi-rooted teeth. Additionally, the evaluation method utilized in this study, involving sectioning and direct measurement of sections with a digital caliper, while providing valuable data, may not fully capture the complexity of radicular anatomy. Despite the use of an ultra-thin silicon carbide separator disc, tissue loss during sectioning could potentially affect the results. Techniques such as micro-CT offer higher resolution and three-dimensional analysis, which could yield more comprehensive insights into radicular dentin thickness. Furthermore, the study's reliance on extracted teeth for analysis may not fully represent in vivo conditions as factors such as occlusal forces and periodontal support can influence radicular dentin thickness. As it is not always possible to adapt the results of extracted teeth to in vivo conditions, further clinical studies in the field are required.

## Conclusions

In conclusion, the study revealed several key findings regarding radicular dentin thickness. Significant variations were observed based on both the level of the root and the surface of the dentinal wall, with buccal and lingual surfaces generally exhibiting greater thickness compared to mesial and distal surfaces. Furthermore, certain tooth subgroups, such as maxillary central, maxillary, and mandibular canine teeth, demonstrated notably higher radicular dentin thickness. Additionally, a consistent trend of decreased thickness toward the apical region was observed. These findings underscore the complexity and variability of radicular dentin thickness across different teeth and root levels, highlighting the importance of such considerations in dental diagnostics and treatment planning.
